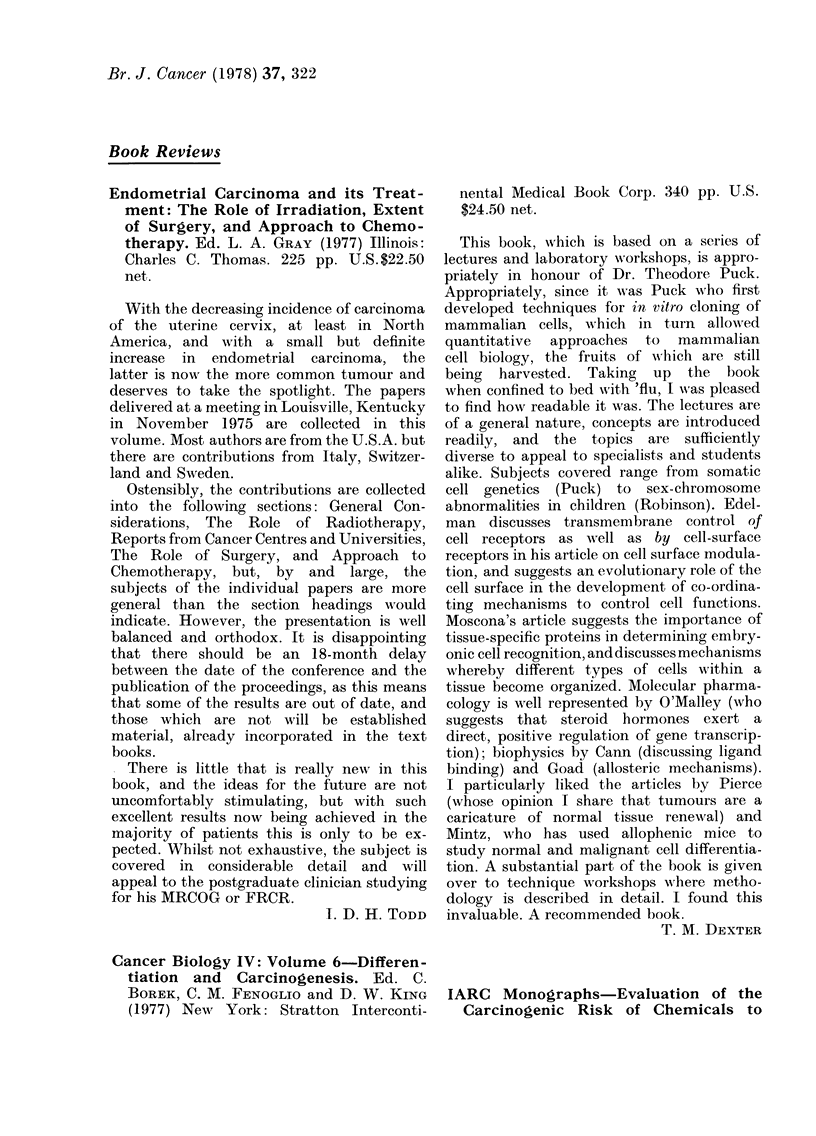# Cancer Biology IV: Volume 6—Differentiation and Carcinogenesis

**Published:** 1978-02

**Authors:** T. M. Dexter


**Cancer Biology IV: Volume 6—Differentiation and Carcinogenesis.** Ed. C. BOREK, C. M. FENOGLIO and D. W. KING (1977) New York: Stratton Intercontinental Medical Book Corp. 340 pp. U.S. $24.50 net.

This book, which is based on a series of lectures and laboratory workshops, is appropriately in honour of Dr. Theodore Puck. Appropriately, since it was Puck who first developed techniques for *in vitro* cloning of mammalian cells, which in turn allowed quantitative approaches to mammalian cell biology, the fruits of which are still being harvested. Taking up the book when confined to bed with 'flu, I was pleased to find how readable it was. The lectures are of a general nature, concepts are introduced readily, and the topics are sufficiently diverse to appeal to specialists and students alike. Subjects covered range from somatic cell genetics (Puck) to sex-chromosome abnormalities in children (Robinson). Edelman discusses transmembrane control *of* cell receptors as well as *by* cell-surface receptors in his article on cell surface modulation, and suggests an evolutionary role of the cell surface in the development of co-ordinating mechanisms to control cell functions. Moscona's article suggests the importance of tissue-specific proteins in determining embryonic cell recognition, and discusses mechanisms whereby different types of cells within a tissue become organized. Molecular pharmacology is well represented by O'Malley (who suggests that steroid hormones exert a direct, positive regulation of gene transcription); biophysics by Cann (discussing ligand binding) and Goad (allosteric mechanisms). I particularly liked the articles by Pierce (whose opinion I share that tumours are a caricature of normal tissue renewal) and Mintz, who has used allophenic mice to study normal and malignant cell differentiation. A substantial part of the book is given over to technique workshops where methodology is described in detail. I found this invaluable. A recommended book.

T. M. DEXTER